# Comment on: “A Machine Learning Approach to Concussion Risk Estimation Among Players Exhibiting Visible Signs in Professional Hockey”

**DOI:** 10.1007/s40279-025-02211-8

**Published:** 2025-04-07

**Authors:** Maximilian Klemp, Robert Rein

**Affiliations:** German Sport University Cologne, Cologne, Germany

To the Editor,

We are writing in response to the article titled *“A Machine Learning Approach to Concussion Risk Estimation Among Players Exhibiting Visible Signs in Professional Hockey”* by Bruce et al., recently published in *Sports Medicine* [1]. We commend the authors for their valuable contribution to the field of concussion risk assessment using machine learning. The authors developed three predictive models—a conditional inference tree, a random forest, and a logistic regression model—using data from 1563 unique events, 183 of which were later diagnosed as concussive. The models reported by the authors show strong discrimination ability, as reflected in AUROC values of 80.2, 81.8 and 82.2 for the conditional tree, random forest, and logistic regression models, respectively. The authors convincingly show that including personal concussion history improves model discrimination. However, we would like to highlight several concerns regarding the performance of these models, especially in the context of clinical decision-making.

The terms *risk model* and *likelihood of concussion* used by Bruce et al. [1] suggest that a probabilistic prediction of the target variable is intended. This aligns with a clinical approach, where estimating the probability of concussion is paramount. While the model choice and training support probabilistic predictions, the evaluation process seemingly overlooks this aspect. In clinical risk prediction models, performance is typically evaluated through two key characteristics: discrimination and calibration [2]. Discrimination refers to the ability of the model to distinguish between positive and negative cases, i.e. whether the model discriminates between cases with greater incidence rates compared to cases with lower incidence rates. As is often the case, discrimination is assessed by Bruce et al. using the area under the receiver operator characteristics curve (AUROC or AUC). One important assumption of this procedure is that sensitivity and specificity are of equal importance. However, whether this is the case with respect to concussions is probably debatable. Calibration in turn, assesses the agreement between the estimated risk and the observed relative occurrence of the event [3]. A well-calibrated model assigns low probabilities to groups with few positive cases and high probabilities to groups with many. Despite the TRIPOD (Transparent Reporting of a multivariable prediction model for Individual Prognosis Or Diagnosis) guidelines recommending the reporting of calibration in prediction modeling studies [4], calibration is far less commonly assessed than discrimination, as found in systematic reviews [5–7]. This oversight is problematic, as poor calibration can lead to misleading predictions [8].

One of the key issues regarding model performance stems from the often-overlooked fact that models can exhibit poor calibration despite good discrimination. For instance, even if a model correctly classifies patients, its risk estimates may be too extreme (too high for positive cases and too low for negative ones) or too conservative (closer to the overall base rate). Such errors make risk estimates unreliable, which can lead to incorrect, potentially harmful decisions [8]. In Bruce et al.’ case, poor calibration of their risk models could either expose players to unnecessary risk or undermine confidence in the model. If predicted probabilities are too conservative, actual cases of concussion could be missed due to underestimation. Conversely, if the predictions are too extreme, concussion risk might be overestimated, leading to excessive, unnecessary off-field examinations. This could ultimately erode trust in the model from coaches and medical staff. Therefore, ensuring proper calibration is crucial when risk models are intended for clinical decision-making.

The problem is exacerbated when using highly flexible machine learning models, which are at an even higher risk of miscalibration [9, 10]. Studies have reported that certain algorithms tend to produce probabilistic predictions disproportionately skewed towards 0 and 1 [11]. This bias is particularly concerning when the binary target variable shows a considerable class imbalance, as in the current study, where concussions occur in only 12% of events (183 concussions out of 1563 events). The issue of miscalibration in machine learning models has even prompted the development of distinguished calibration techniques to adjust model outputs [12–14]. Given this, calibration should not be optional when machine learning models provide probabilistic predictions; it is a necessary step in performance evaluation, especially in clinical risk models used for decision-making.

Several approaches exist for assessing calibration [3], with the most common being the evaluation of the so-called calibration curve [15]. Calibration curves can be assessed both graphically and statistically through the intercept and slope [16]. Therefore, it is good practice to present the calibration curve when documenting a risk model, as it allows for a more comprehensive evaluation of the model’s reliability beyond its discrimination performance. We strongly encourage Bruce et al. to include the calibration curve for their concussion risk model, as this would enhance its utility for practitioners.

## Declarations

### Data availability

No data has been used for this letter.
